# Ovariotomy

**Published:** 1864-04

**Authors:** 


*Ovariotomy.*

Thanks in no small measure to the indefatigable and restless opposition of Dr. Robert Lee, ovariotomy, which had for some time been one of the most successful and important of great operations has also become the most illustrious and renowned. Opposition is the touchstone of truth; and the repeated onslaughts of Dr. Robert Lee have in each case brought down statistics so full and triumphant from Tyler Smith, Baker Brown, Clay, Wells, and others, that the case in favor of the operation has long since been thoroughly established. Here is a disease hopeless, fatal, and remorseless—held till lately to be incurable and deadly: on the other hand, an operation which Mr. Baker Brown has performed fifty-three times during the last twelve years with twenty-nine recoveries, the improvements in the operation being so great that of the last thirty-one operations, there have been only ten deaths. Dr. Tyler Smith has performed it recently in fifteen cases, of which twelve have recovered. Mr. Spencer Wells has operated in fifty-five cases, with thirty-seven recoveries; and of his last twenty cases, eighteen recovered. It is astonishing that any voice should be raised to carp at an operation which has been practised with results so triumphant.—*Lancet*, 1863

